# Supplementary material for: Identification of photoperiod-induced specific miRNAs in the adrenal glands of Sunite sheep (Ovis aries)
Source: Front Vet Sci. 2022 Jul 22;9:888207. doi: 10.3389/fvets.2022.888207 (PMC9354845; doi:10.3389/fvets.2022.888207)
Supplement: Supplementary file 1 [file Data_Sheet_1.ZIP › Supplementary file/miRDeep2 file.zip!/miRDeep2 file/Novel_Structure/SPLP42A1/Novel_351.pdf]

Provisional ID	:	NC_019484.2_32508
Score total	:	1.4
Score for star read(s)	:	-1.3
Score for read counts	:	0
Score for mfe	:	1.1
Score for randfold	:	1.6
Score for cons. seed	:	
Total read count	:	51
Mature read count	:	51
Loop read count	:	0
Star read count	:	0

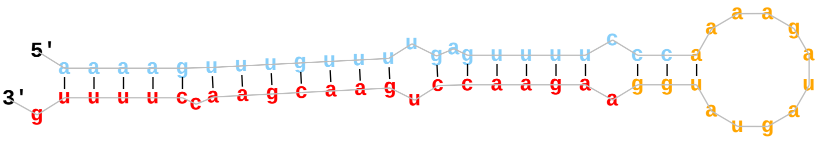

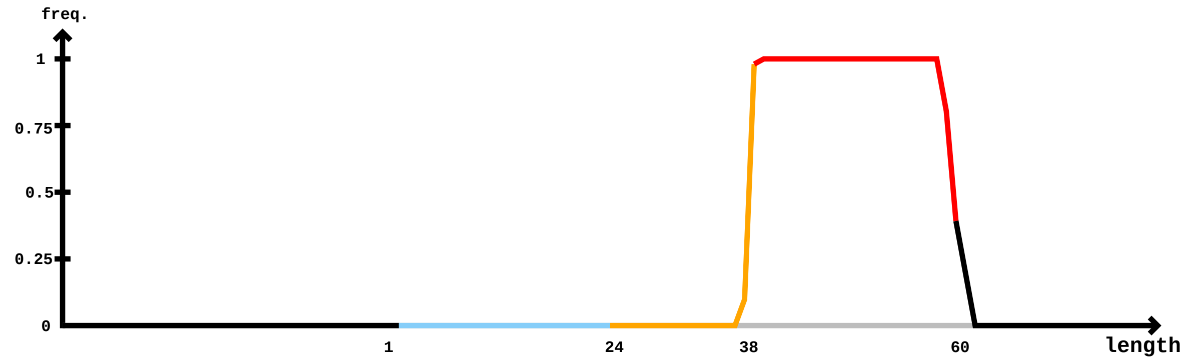

Star Mature

```
5'- ucuguuguggaucaccuuauaucggguuggccgaaaaguuuguuuugaguuuucccaaaagauaguauggaagaaccugaacgaaccuuuuggccaucucaacaaauacuua   -3'    exp
    ..(((((.((((.((((......)))).((((((((.((((((((.(.(((((.(((..........))).))))))).)))))))).)))))))))))))))))........    reads    mm    sample
    .....................................................................gaagaaccugaacgaaccuuu......................    2        0     seq
    .....................................................................gaagaaccugaacgaaccuuuu.....................    3        0     seq
    ......................................................................aagaaccugaacgaaccuuu......................    8        0     seq
    ......................................................................aagaaccugaacgaaccuuuu.....................    17       0     seq
    ......................................................................aagaaccugaacgaaccuuuug....................    17       0     seq
    ......................................................................aagaaccugaacgaaccuuuugg...................    3        0     seq
    .......................................................................agaaccugaacgaaccuuuu.....................    1        0     seq
```
